# Re-establishment of the digestive lumen in a postesophagectomy anastomotic atresia under endoscopic ultrasound guidance

**DOI:** 10.1055/a-1944-9077

**Published:** 2022-10-14

**Authors:** Zheng Zhang, Haiying Zhao, Zhen He, Shutian Zhang, Peng Li

**Affiliations:** Department of Gastroenterology, Beijing Friendship Hospital, Capital Medical University, Beijing, China

A 71-year-old man was referred to our department with a post-esophagectomy anastomotic atresia. Nine months previously, he had undergone transhiatal esophagectomy for esophageal intramucosal squamous carcinoma. Frustratingly, he developed a serious post-esophagectomy stenosis. Several esophageal bouginage and stent procedures failed to stop the progressive stenosis. Finally, a jejunal nutrition tube was placed for feeding, piercing the epigastric skin.

After the large amount of retained fluid had been suctioned, the upper esophagus appeared to be blocked and a guidewire could not be advanced under esophagogastroduodenoscopy guidance ( Fig. 1 ). Endoscopic ultrasound-guided fine-needle aspiration (EUS-FNA) was performed to reconnect the esophagus and stomach ( Fig. 2 a ). Reverse-direction transnasal gastroscopy from the jejunal fistula and digital subtraction angiography were used to monitor the process ( Fig. 2 b ). A zebra guidewire was then placed into the channel to guide the subsequent dilation with a cystotome and placement of a stent. After removing the stent 2 months later without complications, the reopening of the esophageal lumen was confirmed to have been successful ( Fig. 3 ). All the procedures are shown in Video 1 .

**Fig. 1:**
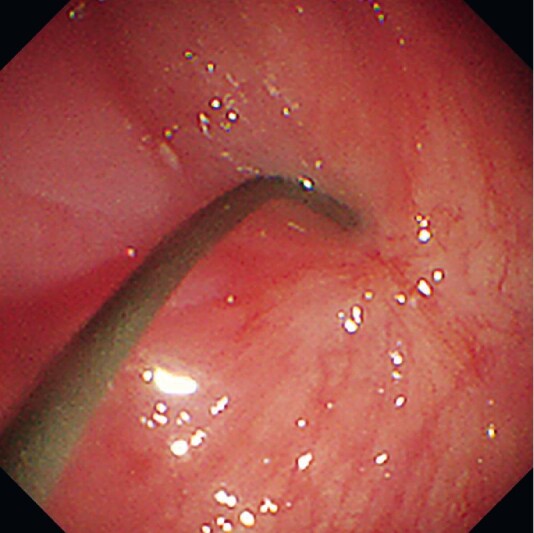
The upper esophagus was blocked and a guidewire could not be advanced.

**Fig. 2:**
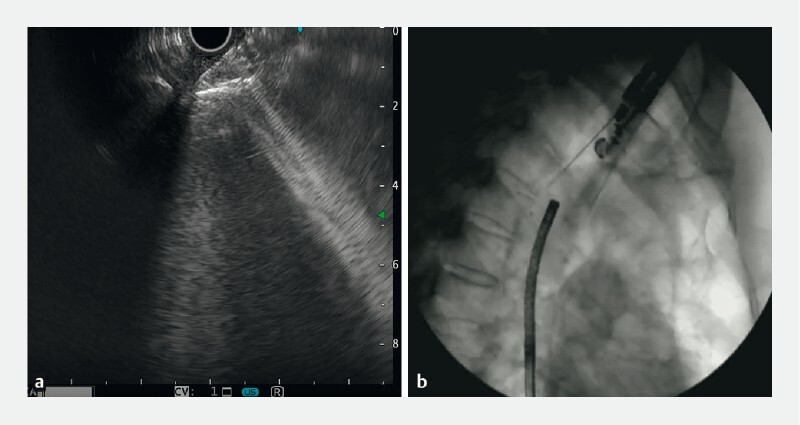
Endoscopic ultrasound (EUS)-guided fine-needle aspiration was performed to reconnect the esophagus and stomach. **a** EUS guidance. **b** Digital subtraction angiography guidance.

**Fig. 3:**
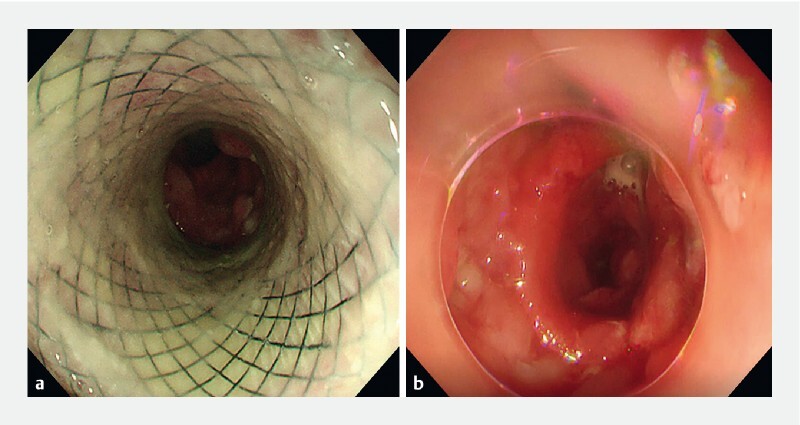
Esophagogastroduodenoscopy 2 months later. **a** The fully patent lumen-apposing metal stent. **b** The anastomotic stoma after stent removal.

**Video 1** Re-establishment of the digestive lumen in a post-esophagectomy anastomotic atresia. Up to three monitors including endoscopic ultrasound, digital subtraction angiography, and reverse-direction transnasal gastroscopy from the jejunal fistula were used to supervise and guide the whole procedure.


Anastomotic atresia developing from severe post-esophagectomy stricture has not been reported previously 1 2 . This case presents a novel way of re-establishing the digestive tract lumen under EUS guidance for anastomotic atresia, suggesting that EUS-FNA could play a greater role in the interventional therapy of digestive tract atresia 3 .

Endoscopy_UCTN_Code_TTT_1AS_2AB

## Notes

Endoscopy E-Videos

https://eref.thieme.de/e-videos
*Endoscopy E-Videos* is an open access online section, reporting on interesting cases and new techniques in gastroenterological endoscopy. All papers include a high quality video and all contributions are freely accessible online. Processing charges apply (currently EUR 375), discounts and wavers acc. to HINARI are available. This section has its own submission website at https://mc.manuscriptcentral.com/e-videos
